# Traditional Medicine Beliefs and Practices among Caregivers of Children under Five Years—The Child Health and Mortality Prevention Surveillance (CHAMPS), Western Kenya: A qualitative study

**DOI:** 10.1371/journal.pone.0276735

**Published:** 2022-11-02

**Authors:** Sarah Hawi Ngere, Victor Akelo, Ken Ondeng’e, Renee Ridzon, Peter Otieno, Maryanne Nyanjom, Richard Omore, Beth A. Tippett Barr

**Affiliations:** 1 Kenya Medical Research Institute, Center for Global Health Research, (KEMRI-CGHR), Kisumu, Kenya; 2 Centers for Disease Control and prevention (CDC), Nairobi, Kenya; Teesside University, UNITED KINGDOM

## Abstract

**Background:**

Approximately 80% of the population residing in sub-Saharan Africa relies on Traditional Medicine (TM). However, literature on factors motivating the use of TM for children under the age of five in these settings is limited. Such information can guide policy formulation for integration of TM into mainstream health care services. This study aimed to describe the motivation on use of TM among caregivers of children residing in rural and urban communities in western Kenya.

**Methods:**

The socio-behavioral sciences (SBS) arm of the Child Health and Mortality Prevention Surveillance (CHAMPS) program in western Kenya, conducted a cross-sectional qualitative study in Manyatta—an urban informal settlement located in Kisumu town and Karemo—a rural setting in Siaya County. We performed 29 in-depth interviews, 5 focus group discussions and 11 semi-structured interviews with community representatives (n = 53), health workers (n = 17), and community leaders (n = 18). All the participants were purposively sampled. We performed thematic analysis using both inductive and deductive approaches. Data management was completed on Nvivo 11.0 software (QSR International, Melbourne, Australia).

**Results:**

Our findings reveal that some caregivers prefer TM to treat some childhood diseases. Use of TM was informed by illness beliefs about etiology of disease. We observed an appreciation from the study participants that malaria can effectively be treated by Conventional Medicine (CM) while TM was preferred to treat measles and diseases believed to be associated with supernatural etiology such as witchcraft, evil spirit or breaching cultural taboos. TM was also used in instances where CM failed to provide a diagnosis or when CM was ‘slow’. TM in such cases was used as a last resort.

**Conclusion:**

We observed varied beliefs that motivate caregivers’ choice of TM use among children in western Kenya. It is therefore crucial to consider perceptions and socio-cultural beliefs about illnesses when formulating interventions that are geared towards child health.

## Introduction

Worldwide, the demand for use of Traditional Medicine (TM) has increased and remains important in the provision of therapy and healthcare [1–3]. According to the World Health Organization, TM is comprised of health practices, approaches, knowledge and beliefs that incorporate plants, animal and mineral based medicines, spiritual therapies, manual techniques and exercises [4]. These are applied as single or combined therapies [5]. It has been estimated that 80% of the world population relies on TM to meet their health care needs [6]. TM use in developing countries is often attributable to its accessibility and affordability. It is entrenched within wider belief and cultural systems [4,7]. TM is an important part of health care provision in many African countries where it is used to treat a wide range of illnesses [4,5]. It may be used in combination with the conventional therapy, particularly for chronic, unexplained or recurrent conditions as well as those that defy conventional scientific treatment that may be attributed to attacks by evil spirits, spell-casting and witchcraft [8]. Spiritual healing is the second most popular TM after herbal treatment and is mostly used to treat unexplained conditions [8–11].

There is evidence that for limited conditions such as diarrhea, dehydration and infantile colic, herbal medications can be effective [12]. An increasing and high prevalence of use has been documented among children with chronic illnesses such as cancer and gastroenteritis and other diseases such as hand and foot disease, malaria and pain during teething [11–15]. In sub-Saharan Africa (SSA), malaria, pneumonia and diarrhea remain the major cause of preventable deaths among children under five years of age [16]. Fever, convulsions and fatigue are symptoms common to these illnesses as well as to multiple other conditions. At times these symptoms have been attributed to supernatural causes such as witchcraft, evil spirits and breaching taboo [17,18].

Response to illness depends majorly on the individual perceptions of cause of the illness [19,20]. The beliefs about the causes of illness can result in customs and practices that can adversely affect illness outcome [21].

Child mortality in Kenya is high with substantial disparities across regions despite momentous effort by the government of Kenya to ensure access to primary healthcare [22]. There are concerns around ubiquitous use of TM because of issues including issues of delay in seeking appropriate care, adverse effect, efficacy, safety, dosing, qualification and licensing of providers, and proper use of products of assured quality. Moreover, parents and caregivers who initiate traditional therapies may fail to disclose this to the medical practitioner resulting in missed opportunity to limit any potential interaction or adverse effects of TM with conventional medicine (CM) [23–26]. The non-disclosure is due to stigmatization caused by poor perceptions towards use of TM. This is due to the over-riding power of CM undermining use of TM [27]. It is therefore inherent to understand childhood illness beliefs and caregiver’s health seeking behavior.

The Child Health and Mortality Prevention Surveillance (CHAMPS) program is set to provide accurate, complete, timely and reliable data on the likely causes of deaths in children under five years of age and inform interventions aimed at reducing the burden in selected sites in 7 diverse countries in SSA (South Africa, Mali, Mozambique, Kenya, Sierra Leone, Ethiopia) and Asia (Bangladesh) [28]. Due to complexities associated with religious beliefs, cultural norms and ethical questions around medical research, the social and behavioral sciences (SBS) arm of CHAMPS program was constituted per site. The main aim was to explore practicality, implementation and acceptability of conducting minimally invasive tissue sampling from which this study was conducted. CHAMPS SBS formative research sought to understand general factors associated with feasibility of CHAMPS surveillance. Approximately 16% of caregivers who were enrolled in CHAMPS-Kenya for minimally invasive sampling (MITS) reported to have used herbs as a treatment course for their deceased child (unpublished CHAMPS data). Despite documented evidence of high prevalence of use among adult populations, there is paucity of literature on prevalence of use of TM among children under five years of age in SSA.

This study seeks to explore the prevailing TM rationalities for use in childhood illnesses for children under five years in Western Kenya.

## Materials and methods

### Study setting

The study was conducted in Manyatta (urban informal settlement) and Karemo (rural) Health Demographic Surveillance (HDSS) sites located in Kisumu and Siaya counties, respectively. The HDSS infrastructure provides longitudinal population-based demographic information and data on the burden of diseases [29]. Jaramogi Oginga Odinga Teaching and Referral Hospital, the largest public referral hospital in the region, is located in Manyatta while Siaya County Referral Hospital is located in Karemo HDSS area.

### Study design

The design of the formative phase of socio-behavioral research of CHAMPS surveillance and CHAMPS surveillance methods have been published elsewhere [28]. In brief, the current study employed a qualitative study design using a mix of ethnography and phenomenological approaches. The methods for this formative research involved a combined phased-approach of semi-structured interviews (SSIs), key informant in-depth interviews (KIIs), focus group discussions (FGDs) and participant observations. This multi-method approach was to facilitate data triangulation needed to validate information collected across different data sources. For this paper we report KII, SSI and FGD results only. The SSI guide was open with a pre-determined format and sequence, but allowing some flexibility in the way the topics were addressed by both interviewer and respondent. Despite the SSI guide having some degree of structure, the respondent was encouraged to expound on ideas rather than giving “yes” or “no” responses. The KII guides were broad, less structured, open-ended sets of verbal questions based on an interview topic guide. FGD guides were semi-structured thus giving flexibility to participants to elaborate views and opinions. All the interview guides were developed in three languages (English, Swahili and Dholuo). The stakeholders for SSI, KII and FGDs were caregivers, healthcare workers, traditional birth attendants, traditional healers, religious leaders and community leaders. The KII participants were those involved in the events around death in order to gain an in-depth understanding of cultural, social and religious norms. SSI and FGD participants were conversant with the community culture by virtue of being residents but did not have in-depth understanding of the topic area. They were only asked of their views, concerns and expectations of individuals representing entities or institutions that implement or conduct MITS.

### Study participants

The study enrolled a total of 88 male and female participants of above 18 years of age residing in Manyattta location, Kisumu county and Karemo division, Siaya county. Participants were selected purposively for the formative phase of socio-behavioral research arm following the principle of maximum variation sampling. Maximum variation sampling strategy has the advantage of optimizing the variety of participants to answer the research question [30]. Participants from the socio-behavioral arm of CHAMPS program were placed into six different categories. Participant category was selected in accordance to a pre-determined sample size from the SBS protocol [31]. The study categories were as follows; community representative category included; religious leaders (Christian and Islam representative), caregivers and community leaders (opinion leaders, chiefs, assistant chiefs and village elders). Health care providers included; healthcare workers, traditional birth attendants (TBA) (traditional midwives), and traditional healers (traditional medicine practitioners/herbalists). Specifically, we performed 29 in-depth interviews, 5 focus group discussions and 11 semi-structured interviews with community representatives (n = 53), health workers (n = 17), and community leaders (n = 18). All participants were only interviewed once.

### Data management & analysis procedures

FGDs were conducted by one moderator and a note taker while SSIs and KIIs were done by only one interviewer. Audio recorded discussions were transcribed verbatim and translated from Dholuo or Swahili to English. *Nvivo version 11*.*0*, a qualitative data analysis program, was used to organize the data and code themes from the transcribed discussions. We adopted a sequential approach to analyzing the data by initially applying a deductive approach followed by an inductive approach to make full sense of the data. Deductive coding involved developing codebook from the research questions and a list of *priori* categories were generated based on previous research. The indicative phase involved an iterative process; going through the data back and forth assigning codes to paragraphs or segments of texts as concepts unfold through a recursive process using the research questions as the lenses. The lead author interpreted the themes and identified what best depicted the topic of this manuscript, summarized the data descriptively and wrote up the final report capturing quotes representing the main themes. Quotes were selected on the basis of their clear representation of themes.

### Ethical considerations

The current study was part of Child Health and Mortality Surveillance (CHAMPS) Kenya study which was approved by the KEMRI Ethics Review Committee (KEMRI Protocol # 3313). In brief, verbal informed consent was obtained from each participant in the study. Verbal consent was used because the study was of minimal risk to the participant. All audio recordings were de-identified and stored in laptops and central storage device and deleted from audio recorders. All persons involved in data collection/entry/transcription were trained in the handling of confidential information, and in good data management practices. For data security, laptops holding data were protected by strong passwords for logging in users; the storage devices were kept in lockable cabinet and were only accessed by the authorized staffs.

## Results

Table 1 presents the socio-demographic characteristics of the study participants. A total of 88 participants were interviewed, 51 (57.9%) females and 37 (42%) males. A total of 38 (43%) had at least secondary education or higher with majority 80 (91%) being Christians.

**Table 1:** Socio-demographic characteristics of study participants (n = 88).

	Caregivers	Health workers	Religious leaders	Traditional Birth attendant	Traditional healer	Community leaders	Total
**Gender**							
Female	33	9	1	3	1	4	51(57.9%)
Male	20	4	6	0	0	7	37(42%)
**Age**							
<30	26	3	0	0	1	0	30(34%)
31–49	19	8	3	0	0	4	34(38.6%)
50+	8	2	4	3	0	7	24(27%)
**Highest level of education**							
None	1	0	0 0	0	0		1(0.01%)
Upper Primary	18	0	5	1	0	4	28(31.8%)
Lower Primary	5	0	0	1	0	0	6(6.8%)
Some Secondary	14	0	1	0	0	0	15(17%)
Secondary	7	0	0	1	1	3	12(13.6%)
College and above	8	13	1	0	0	4	26(29.6)
**Source of income**							
Formal employment	1	13	4	0	0	3	21(23.9%)
Self-employment	32	0	3	3	1	4	43(48.9%)
Non formal employment	6	0	0	0	0	4	10(11.3%)
Other	14	0	0	0	0	0	14(15.9%)
**Religion**							
Christian	48	12	6	3	1	10	80(90.9%)
Muslim	3	0	1	0	0	0	4(4.6%)
Other	2	1	0	0	0	1	4(4.6%)

### Thematic findings

We identified three main themes: rationale of use of TM (theme 1), factors motivating disengagement from TM (theme 2) and trusted sources (theme 3). Table 2 summarizes the thematic areas as well as the major findings for each area.

**Table 2:** Summary of thematic areas and key findings.

Themes	Summary of major findings
**Rationale of use of TM.** **Sub themes:** **i. Illness beliefs** **ii. Etiology of disease** This theme examines the rationalities of using TM among children in our context. Local beliefs regarding illness, TM and CM use are addressed.	• Beliefs that some diseases cannot be treated in the hospital. Measles was believed to be only treatable with TM • Diseases can be caused by evil spirits and witchcraft and breaching taboo • Caregivers report that some diseases/conditions can be managed from use of TM. • If traditional illness are treated by injection then the child would die
**Factors motivating disengagement from CM.** **Sub themes:** **i. When CM fails to provide a diagnosis** **ii. When CM is ‘slow’** This theme examines the motivating factors that contribute to use of TM. Disengagement from CM may be attributed to cultural beliefs. Cultural beliefs such as witchcraft are addressed.	• There is a belief that illnesses that do not respond to CM or when diagnostics cannot identify the cause of an illness, then the illness is caused by supernatural forces such as witchcraft and the devil. • When illness is slow to respond to CM there is a common belief that TM will work much faster in curing the illness. • Switching from CM to TM was done when caregivers felt that CM was unhelpful.
**Trusted sources** **Sub theme:** **i. Treatment recommendation** This theme examines the role of family and the community in child health.	• Treatment decision making is an inherently social process where others in the community also give advice on treatment for childhood illness. • Neighbors are consulted for child illness and they assist in making decisions for the best healthcare choice • Treatment recommendation to seek treatment from a traditional healer can either be successful or cause undesirable outcome

## Rationale for use of TM

### Illness beliefs

#### Some diseases cannot be treated in the hospital

The majority of respondents believed that measles cannot be treated with CM, while malaria must be treated urgently with CM. Traditional remedies was commonly used to treat measles in the community. Herbal concoctions are prepared and administered in different forms.

*I’d say it depends on the sickness that you are seeking treatment for*. *Let’s say for children you’ll look at the type of sickness they have*. *It could be measles; most people always go for traditional remedies*. *You make the child sniff marijuana (bhang) and it (measles) goes away and if it is malaria you will either rush to Russia or District (hospitals)*.

*(Male*, *caregiver >30 years)*

Respondents report that some diseases/conditions can be effectively managed from use of TM.

*When I think of illnesses that may make people not go to the hospital*, *we have the measles which can kill a child*. *A child would diarrhea a lot*. *I usually see older women—and even if you go to Kibuye (market in Kisumu)—there are herbs which they mix that are used to bath a child and there are also others which they are given to drink*. *Later you find rashes appearing on the body and it is said that that thing is leaving the body*.

*(Male*, *Religious leader between 31–49 years old*)

There is a common belief that some childhood diseases should not be treated using injection therefore when a child is taken to the hospital and injected they will die. Because of this known belief in the communities of Kisumu and Siaya most caregivers would seek TM if malaria results are negative.

*… if they do not find malaria they can use other drugs but if they make a mistake of injecting then the child dies and that contributes to many deaths among children*.

*(Female*, *traditional healer 50+ years)*

### Etiology of diseases

#### Diseases caused by evil spirits and witchcraft

Respondents report that there are instances when TM is used as a form of protection against evil spirits that may cause diseases. Women who have lost children are considered unclean in this setting and therefore need cleansing in order to be integrated back into the community after the loss of a child. If this is not done, young children and pregnant women are at risk of being affected by the evil spirit which will lead to expectant mothers losing their unborn children or a young child getting sick and dying.

*If you meet with her (mother who has lost a child)*, *you will be told that you met a mother who lost a child and she has affected you with an “evil shadow’ so you should be gathering dirty leaves at a cross road then you burn and give to the child to stop diarrhea*.

*(Female*, *traditional healer 50+ years*)

Quite a number of respondent’s report that there are individuals in the community capable of making a young child fall ill through their ‘evil eye’. Therefore, they have to be treated with traditional form of medicine.

*There is also the bewitching or evil eye*. *You find one “flushing” your child (giving them the evil eye) when you take the child to hospital and they are injected then they will definitely die so the child has to be taken somewhere else (to seek services of a traditional healer)*.

*(Male*, *caregiver >30 years*)

The community believes that there are instances where a child may fall ill because of his/her mother’s wrong doing mostly when the mother goes against taboo in the community.

*There are illnesses that can ail a child that is called ‘Chira’ (illness due to breaching taboo)*. *The child can get it from the mother if she does something wrong (breaching taboo) then they would look for ‘manyasi’ (herbal concoction that remedies the effects of doing something against cultural norms)*.

*(Male*, *caregiver50+ years*)

### Factors motivating disengagement from CM

#### When CM fails to provide a diagnosis

Respondents report that when medical treatment is sought and all test results come out negative, this means that the sick child has been bewitched and therefore requires prayers or treatment by a traditional healer.

*The diseases which one is prayed for are the ones that someone complains of a sudden headache and it just aches very fast (explains the increasing intensity of the headache) and when he goes (probably to a health facility) to test for malaria they don’t find it so you may think that there is something earthly troubling him so you take him to the faith healers*. *Or you go to the “clever” people such as the traditional healers and you find that most people with such diseases lose their lives*.

*(Male*, *community leader 50+ years*)

These diseases as some respondent’s report may be coming from the devil (Satan) and consequently may not be treatable using CM hence the need to seek TM.

*There are diseases that come from Satan*. *That is if a person first goes to the hospital they will do an x-ray and find nothing they don’t find an illness*. *They can do other things and find nothing*, *urine test nothing*, *blood test nothing*, *nothing*, *do an ultrasound nothing*. *We have different kind of tests at our hospitals*, *the truth is if they test someone and do not find anything they start to worry and starts asking themselves if they had tested everything even HIV/AIDS and is non-reactive*, *does not have malaria*, *no typhoid and this person feels sick*.

*(Female*, *TBA 50+years*)

#### When CM is ‘slow’

TM was reported to be a better alternative when there is slow or no response to CM. Slow or no response to conventional treatment may result in seeking TM from faith healers and traditional herbalist.

*You cannot know that*. *You know you can take the child for treatment but you do not see any improvement*. *Sometimes you find that the child has fever*, *has diarrhea and vomiting then you go to the hospital and she is tested for malaria and is given a drug and completes the dose*. *And then you go back and explain to the doctor the symptoms and test them again and can change the medication*. *If you go back to the doctor three times*, *then you can have suspicion that maybe it was not a disease but the hands of man (bewitchment) then you can take them for prayers*.

*(Female*, *caregiver between 31–40 years*)

Respondents report that the community believes that some illnesses respond faster to TM compared to CM. They acknowledge that these illnesses will eventually be treated by CM but herbal medication will act faster and therefore the sick person will get better faster.

*There is also “amoeba” this disease that makes (people) to diarrhea sticky things like mucus*. *If it reaches a stage when one diarrhea mucus*, *that is very strong*, *so amoeba has its own medicine also*, *it can be treated in the hospital but it will take long as compared with the herbal medication*. *So the herbalist will treat it faster than the hospital because their drugs go direct but the others have passed through processes and that makes it slow*, *we don’t dispute that hospital treats it but it treats it slower but herbalist treat it very fast if you find the person with the drug*. *It treats it very fast*.

*(Female*, *traditional healer 50+ years*)

### Trusted sources

#### Treatment recommendation

Respondents report that they get TM recommendations from others in the community in cases where the hospital had failed to provide a clear diagnosis.

*When I gave birth to my first born*, *my child had symptoms such as fever and sweating*. *I took him to the hospital and when he was tested he was found not to be having malaria*. *When I came back with him I was not seeing any changes and a neighbor told me that “your child might have been flushed” (bewitched) and she took me to someone*. *When we went to that person (witchdoctor) he told me that my child had been bewitched even before I could say what had taken me there*. *There is something he did and he removed some things from the child and from there the child was alright*.

*(Female*, *caregiver >30 years*)

Sometimes community members would be advised to use TM first to treat an ailing child before using medicine given at the hospital.

*So you have two medicines*, *the traditional one and the one from the doctor*. *So when I go back home*, *you find you are being advised by the neighbor to first administer the traditional one*. *So you have two different medicines and when you give them to the child*, *they end up not working on the body of the child and you end up losing the child*.

*(Female*, *caregiver50+ years*)

## Discussion

Our results revealed that the rationale for use of TM are varied, embedded in cultural beliefs, and dependent on the perceived type of illness and advise the caregivers received from their trusted sources in the community. Respondent report that measles should only be treated with herbal medicine, however, further evidence is needed to explore community beliefs about measles. Similarly in Tanzania, convulsion, a symptom of severe malaria was linked to supernatural causes, a traditional healer was therefore considered to be more appropriate for treating such illness [32]. While reports from other studies reported malaria symptoms to have mystical etiology, we report an awareness by the respondent that malaria should be treated with CM [33,34]. It is however worth noting that malaria negative results were treated with speculations and caregivers would therefore believe that the disease is of supernatural etiology. This may reflect success in interventions that are in place to strengthen caregiver’s awareness of malaria symptoms and danger signs but may inform of a failure of healthcare providers to communicate with caregivers about causes of child illness and treatment course.

In Africa, beliefs about illness etiology influence treatment seeking decisions such that illness with mystical etiology are typically treated using TM [20,21]. Traditional medicine may suffice for common self-limiting diseases but may cause delays in seeking appropriate clinical management for severe diseases such as malaria [26]. Respondent’s perceived child illness to be caused by supernatural agents such as witchcraft, evil spirits, and breach of taboos. Our study reveals a belief that mothers who breach taboo can inflict a child with an ailment. Common belief is that to ‘cure’ these illnesses or prevention of such illnesses would be better addressed by TM, contributing to existing delays in seeking health services, diagnosis and treatment [24,34–36]. Our study reports a common belief that some diseases cannot be treated in the hospital and if drugs are administered using an injection then the child will die. This belief is similar to what was reported in Tanzania [32]. The rationale behind this belief was not determined in either our study or that of Makundi *et al*.

Often, caregivers are apprehensive of childhood illnesses and would associate them with bewitchment when there is slow or no response to CM or when hospitals cannot provide a diagnosis. It was not always that caregivers imply that slow response was caused by witchcraft but inefficacy of CM to treat the illness. Failure of healthcare providers to communicate effectively with caregivers about the cause of the illness and what to expect from the treatment may be the reason of switching from CM to TM. A study in South Africa corroborates with our findings that caregivers consulted traditional healers having experienced formal providers as unhelpful. The switch from CM to TM was not due to belief in bewitchment but out of desperation [37].

Consultation with family members and neighbors regarding child illness is common practice in the African setting. The advice provided may have negative implications on child survival. In our study we report that caregivers are often advised to use TM after consultation with neighbors which is in contrast with previous reports where family members such as grandmothers and mother-in-law were culturally expected to give advice on an illness [28]. This can be attributed to the changes in family structures where families live separately from their extended family particularly in our Manyatta setting which is a peri-urban setup.

Use of TM remain important to the community in managing childhood diseases and has great potential in improving health and wellbeing of people. Hence, there is need for evidence-based standards for quality and evaluation of safety to support regulation.

The strength of our study is that it generated comprehensive in-depth insights of the understanding of rationale of TM use among children under five years of age. It further illuminates the cultural health practices and beliefs of caregivers in western Kenya. A key methodological strength is using both inductive and deductive reasoning for data analysis.

This study is subject to some potential limitations. First, the study did not collect data on religious beliefs to help understand whether any of the observed beliefs could be driven by certain religious norms and economic factors in this setting. Second, the study did not assess prior use of TM and therefore some observations may be hypothetical.

## Conclusion

Our study revealed diverse TM practices and rationale for use among children under five years of age in this setting. These findings also extend the evidence of medical pluralism and the complex care seeking trajectory with potential adverse implications for treatment outcomes among children under five years old. To mitigate the gaps identified in this study, healthcare providers should communicate to caregivers about child illness and treatment course. They should also reassure caregivers and give counselling to abate desperation. Strengthening existing interventions to improve knowledge about danger signs, symptoms and response to childhood illness are encouraged. Engaging mothers, caregivers and their partners to respond promptly to childhood illness by seeking medical attention at the health facilities would reduce severe illness and death. Lastly, there is an urgent need to improve safety and quality standards of TM products to help facilitate regulation and integration into the mainstream health system.
